# Physical activity in post-COVID-19 condition: A cross-sectional study

**DOI:** 10.1371/journal.pone.0357465

**Published:** 2026-09-01

**Authors:** Julia Kopp, Dara Corina Spies Rodriguez, Lisa Künzi, Milo A. Puhan, Jan Sven Fehr, Thomas Radtke

**Affiliations:** Epidemiology, Biostatistics and Prevention Institute, University of Zurich, Hirschengraben, Zürich, Switzerland; La Trobe University - Bundoora Campus: La Trobe University, AUSTRALIA

## Abstract

**Background:**

Post-COVID-19 condition (PCC) is a systemic disease which can negatively influence physical activity (PA). This study aimed to examine objectively measured PA and identify its factors in adults with PCC.

**Methods:**

Cross-sectional analysis of baseline data from a randomised-controlled trial. PA was measured with accelerometry (ActiGraph wGT3X-BT) over eight consecutive days. The primary outcome, mean 24-hour Euclidean Norm Minus One (ENMO), and the secondary outcome, the intensity gradient, were analysed using multivariable linear regression models adjusted for pre-defined covariates.

**Results:**

Among 153 participants, 141 (76.6% female, age range 18−80 years) had valid PA data and were included in the analysis. Median [IQR] time spent in moderate and vigorous PA was 45.9 [32.1–61.2] and 0.16 [0.04–0.6] minutes per day, respectively. The median ENMO was 9.03 [7.2–11.2], indicating low overall PA. In regression models, chronic symptomatic disease other than PCC (β = −1.77, 95% CI = −2.93 to −0.61) and fatigue (β = 0.09, 95% CI = 0.02 to 0.16) were associated with lower mean ENMO, while higher functional capacity was associated with higher mean ENMO (β = 0.14, 95% CI = 0.05 to 0.24). For the intensity gradient, higher education and functional capacity were associated with a more favourable gradient.

**Conclusion:**

Presence of chronic symptomatic disease in addition to PCC, fatigue, functional capacity, and educational attainment were identified as factors of PA. These factors may be relevant for the development of therapeutic strategies to support physical activity; however, causal relationships cannot be inferred from the present study.

## 1. Introduction

Post-COVID-19 condition (PCC) remains a significant burden that can lead to persistent limitations in everyday life [1–3]. The World Health Organization (WHO) introduced the term PCC to include the broad spectrum of symptoms that persist for three months or longer following a severe acute respiratory syndrome coronavirus-2 (SARS-CoV-2) infection and cannot be explained by an alternative diagnosis [4]. The pathogenesis of PCC is considered multifactorial, potentially involving viral persistence, immune dysregulation, coagulation disturbance, chronic inflammation, mitochondrial dysfunction, endothelial dysfunction, and alterations in gut microbiota [5,6]. The most common symptoms are fatigue, dyspnoea, post-exertional malaise (PEM), and concentration difficulties [7,8].

Physical inactivity is a serious global burden that can have far-reaching health consequences. In 2022, nearly one in three adults worldwide were insufficiently active, and this proportion is expected to increase further by 2030 [9]. According to the WHO, physical inactivity in adults is defined as not meeting the recommended levels of at least 150–300 minutes of moderate-intensity aerobic-type activity or at least 75 minutes of vigorous intensity activity per week, along with muscle-strengthening activities on two or more days [10]. Global estimates suggests that physical inactivity accounts for over 13 million disability-adjusted life years lost annually (DALYs), reflecting a considerable burden of non-communicable disease and premature mortality [11], [12]. Furthermore, chronic physical inactivity is associated with reduced muscle mass, strength, and function [13].

Physical activity (PA) may be significantly reduced in people with PCC. Symptoms such as fatigue, PEM, and cognitive difficulties have been reported to be associated with lower levels of PA [14], while PEM is further linked to skeletal muscle abnormalities and impaired oxidative metabolism [15]. While physical inactivity is a known risk factor for chronic diseases [11,12], individuals with PCC may be particularly vulnerable, as their symptoms can persist over extended periods. Prolonged inactivity may create a vicious cycle, where symptoms precipitate inactivity, and inactivity subsequently worsens symptoms, functional decline, and potentially the development of secondary chronic conditions. While most research on PA in PCC relies on self-reported measures, studies using objectively measured PA are scarce. This study aims to 1) characterise objectively measured PA levels in a heterogeneous population of adults with PCC, and 2) to investigate modifiable and non-modifiable factors of PA.

## 2. Methods

### 2.1. Study design and setting

For this cross-sectional study, we used baseline data from a single-centre, placebo-controlled, quadruple-blind, parallel design randomised superiority trial in which participants were randomly allocated to receiving either Pycnogenol^®^ or placebo [16]. Recruitment took place at the University of Zurich (UZH), Switzerland, between 14 June 2023 and 5 July 2024. For this study, we included baseline data of participants who were randomly assigned between June 14, 2023, and August 02, 2024. The trial was approved by the ethics committee of the Canton of Zurich, Switzerland (Kantonale Ethik Kommission Zürich; BASEC-Nr. 2022−01967), the study protocol has been published [16]. Six individuals with lived experience actively contributed to the study planning and offered valuable input regarding outcome measures and the overall organisation of the study visits. The study was performed according to the Declaration of Helsinki and reported according to the STROBE guidelines for reporting cross-sectional studies [17].

### 2.2. Participants

We invited individuals with PCC to participate if they were 18 years or older, spoke German fluently, had no planned changes in medication, and had a confirmed SARS-CoV-2 infection verified by polymerase chain reaction (PCR), rapid antigen test, or a physician’s diagnosis of PCC. Eligible participants were also required to have no untreated comorbidities and report at least one persistent PCC-symptom such as fatigue, cognitive impairment (“brain fog”), dyspnoea, or PEM. We recruited participants through the Altea network, the Long COVID Citizen Science Board, the Facebook group of the patient organisation Long COVID Schweiz, and advertisements placed in public transport across Zurich and its metropolitan area. We also distributed online flyers, mainly targeting hospitals, to raise awareness about the study. All participants signed a written informed consent form before enrolment. Only participants with valid baseline accelerometer data were included in this analysis.

### 2.3. Data sources and measurement

All data for this study were collected at screening and baseline of the PYCNOVID study [16]. Participants completed online questionnaires using the Research Electronic Data Capture (REDCap) and underwent clinical assessments during study visits. PA was measured with a triaxial accelerometer (ActiGraph wGT3X, Pensacola, FL, USA) worn around the hip for eight consecutive days before the baseline visit. The devices were programmed to record raw acceleration with a frequency of 30 Hz. Participants were instructed to wear the devices during waking hours, and to remove them during water-based activities. Questionnaires assessed demographics and medical history (including chronic conditions). Symptom burden was assessed using a 5-point Likert scale (“not bad at all” to “very severe”). Fatigue was measured with the 13-item FACIT-Fatigue, with scores <34 indicating clinically relevant fatigue [18,19]. Severity of dyspnoea, cognitive difficulties, and PEM were assessed using the 5-point Likert scale. We did not assess PEM using a validated instrument, which limits the validity of our evaluation of this complex symptom. In addition, dyspnoea was evaluated with the Chronic Respiratory Questionnaire (CRQ) dyspnoea domain [20,21].

The self-reported health status was measured daily over seven consecutive days with the EQ-VAS (0 = worst imaginable health, 100 = best imaginable health) [22,23]. Mean scores were calculated for participants with ≥4 days of data. Functional capacity was assessed with the 30-s sit-to-stand test (30-s STS) [24].

### 2.4. Potential factors of PA

We predefined a set of variables to be considered as potential factors of PA based on content knowledge and a review of the literature. Data were derived from the screening questionnaires and baseline assessments. These included demographic characteristics (age, sex, and level of education), PCC-related symptoms (PEM and fatigue), physician-diagnosed chronic symptomatic diseases (cardiovascular and metabolic disorders, respiratory diseases, autoimmune and chronic kidney diseases, as well as psychiatric conditions), and functional capacity (30-s STS). Furthermore, we included self-rated health status (EQ-VAS) as an additional factor in a sensitivity analysis. Since the EQ-VAS assesses overall health status on a 0–100 scale (0 = worst imaginable health, 100 = best imaginable health), it likely reflects PCC-related symptom burden, particularly fatigue and PEM, and thus mask PCC-related sequelae. We therefore included the EQ-VAS only in the sensitivity analysis.

### 2.5. Outcomes

The primary outcome was the mean 24-hour Euclidean Norm Minus One (mean 24h ENMO), expressed in milligravity units (mg). This continuous metric, derived from raw accelerometer data, reflects overall PA intensity across a full day, with higher values indicating higher activity levels [25]. The secondary outcome was the intensity gradient (IG). This is a unitless measure describing the slope of the log-log relationship between activity intensity and the time accumulated at each intensity level. Higher (less negative) intensity gradient values indicate a greater proportion of higher-intensity activity, whereas lower (more negative) values indicate activity dominated by lower intensities [25]. In addition, we report the time spent in light, moderate, and vigorous intensity PA using cut-off values from Hildebrand et al. [26,27] and report the number (percentage) of participants meeting PA guidelines by WHO [10].

### 2.6. Sample size

No formal sample size calculation was conducted for this analysis. The sample consisted of all randomised participants from the PYCNOVID trial who had valid accelerometry data. The original sample size for the PYCNOVID trial was based on power calculations related to its primary outcome, as detailed in the trial protocol [16].

### 2.7. Data processing

Raw gt3x files were imported into R using read.gt3x package (version 1.2.0) and processed with the GGIR package (version 3.2.6). GGIR has been described elsewhere [28]. All analyses were performed on a local computing environment. Raw.gt3x files were auto-calibrated to local gravity (1 g ≈ 9.81 m/s²) and converted to 5-second epoch summaries using the ENMO metric, expressed in milligravity units (mg).

Data were summarised across waking hours only, as participants removed the ActiGraph during sleep and water-based activities. PA intensities were classified using ENMO thresholds of 47.4 mg for light, 69.1 mg for moderate, and 258.7 mg for vigorous PA, with activity bouts defined using a 90% adherence criterion [26,27], meaning that at least 90% of epochs within a bout were required to meet the corresponding intensity threshold (e.g., in a 10-minute bout, up to one minute could fall below the threshold).

Activity recordings with a minimum wear time of 10 hours per day with at least three valid weekdays and one valid weekend day were included in the analysis. All analyses were performed using parallel processing to reduce computation time in this large dataset. This refers to the simultaneous processing of multiple files across available CPU cores and does not affect the underlying analytical procedures. Default GGIR calibration procedures were applied, and non-wear time was identified using the built-in raw acceleration–based algorithm.

### 2.8. Statistical analysis

We summarised baseline characteristics and questionnaires using medians and interquartile ranges (IQR) for continuous variables and counts with percentages for categorical variables. In addition, we calculated percent predicted values by dividing each participant’s observed mean ENMO value by the corresponding age- and sex-specific reference value derived from a large population-based German cohort (NAKO) [29], and multiplying the result by 100. The proportions of participants below 100% predicted and below 80% predicted were calculated and are reported descriptively. The 80% predicted threshold was used as a pragmatic clinical benchmark and should not be interpreted as a statistically derived lower limit of normal. Of note, the NAKO reference population was used solely for external descriptive comparisons with our study population and cannot be considered a matched control group. To explore associations between predefined variables and PA (i.e., ENMO and intensity gradient), multivariable linear regression models were built. Separate models were built for each outcome.

## 3. Results

Between June 14, 2023, and July 5, 2024, 170 individuals were screened for eligibility. Among them, 153 met the eligibility criteria and consented to participate. Of those, 141 participants had valid PA data and were included in the final analysis (S1 Table in S2 Appendix, S1 Fig in S1 Appendix. Table 1 summarises participants’ characteristics. Most of the participants were female (76.6%) and the median age was 45. The number and percentage of participants with fatigue (FACIT-Fatigue, score <34), depression or anxiety (HADS, score ≥7), and cognitive impairment (MoCA, score <26) were as follows: fatigue 116 (82.3%); anxiety 55 (39.0%); depression 63 (44.7%); and cognitive impairment 19 (13.5%). In addition, perceived health status (EQ-VAS) was low with a median [IQR] of 48.7 [37.5–64.3].

**Table 1:** Characteristics of study participants with valid accelerometry data.

Characteristics	N = 141
**Female**, n (%)	108 (76.6)
**Age**, years	45.0 [34.0–55.0]
**BMI**, kg.m^-2^	24.4 [21.8–28.5]
**Smoking status**	
Never, n (%)	91 (64.5)
Former, n (%)	40 (28.4)
Current, n (%)	10 (7.0)
**Education level**	
Lower secondary, n (%)	4 (2.8)
Upper secondary, n (%)	40 (28.4)
Tertiary, n (%)	97 (68.8)
**Hospitalised for SARS-CoV-2 infection,** n (%)	6 (4.3)
**Comorbidities**	
Obesity, n (%)	26 (18.4)
Hypertension, n (%)	11 (7.8)
Diabetes, n (%)	3 (2.1)
Cardiovascular disease, n (%)	7 (5.0)
Chronic kidney disease, n (%)	3 (2.1)
Chronic respiratory disease, n (%)	19 (13.5)
Psychiatric disease, n (%)	18 (12.8)
Autoimmune disease, n (%)	7 (5.0)
Other diseases, n (%)	47 (33.3)
**FACIT-Fatigue**	23.5 [17.8–30.0]
**HADS**	
Depression	6.0 [4.0–9.0]
Anxiety	5.0 [3.0–8.0]
**CRQ dyspnoea**	6.0 [5.0–6.6]
**MoCA**	28.0 [27.0–29.0]
EQ-VAS*	48.7 [37.5–64.3]
**Self-reported PCC symptoms**	
Post-exertional malaise, n (%)	132 (93.6)
Fatigue, n (%)	121 (85.8)
Dyspnoea, n (%)	92 (65.2)
Brain fog, n (%)	127 (90.1)
**Practising pacing as treatment strategy**, n (%)	94 (66.7)
**Movement therapy**^**#**^, n (%)	90 (63.8)
**30-s STS**, no of repetitions	15.0 [11.0–19.0]

Data are n (%) or median [IQR] unless otherwise specified; Obesity defined as body mass index > 30 kg.m^-2^; BMI: body mass index; CRQ: Chronic Respiratory Questionnaire; EQ-VAS: EuroQol Visual Analogue Scale; FACIT-Fatigue: Functional Assessment of Chronic Illness Therapy – Fatigue; HADS: Hospital Anxiety and Depression Score; MoCA: Montreal Cognitive Assessment. *Median values were calculated from the mean value from daily EQ-VAS data for each individual participant at baseline. #Movement therapy includes activities such as rehabilitation, walking, and yoga; Missing data: two for 30-s STS, one for FACIT-Fatigue, one for HADS, one for CRQ dyspnoea, and one for self-reported PCC symptoms.

**Fig 1:**
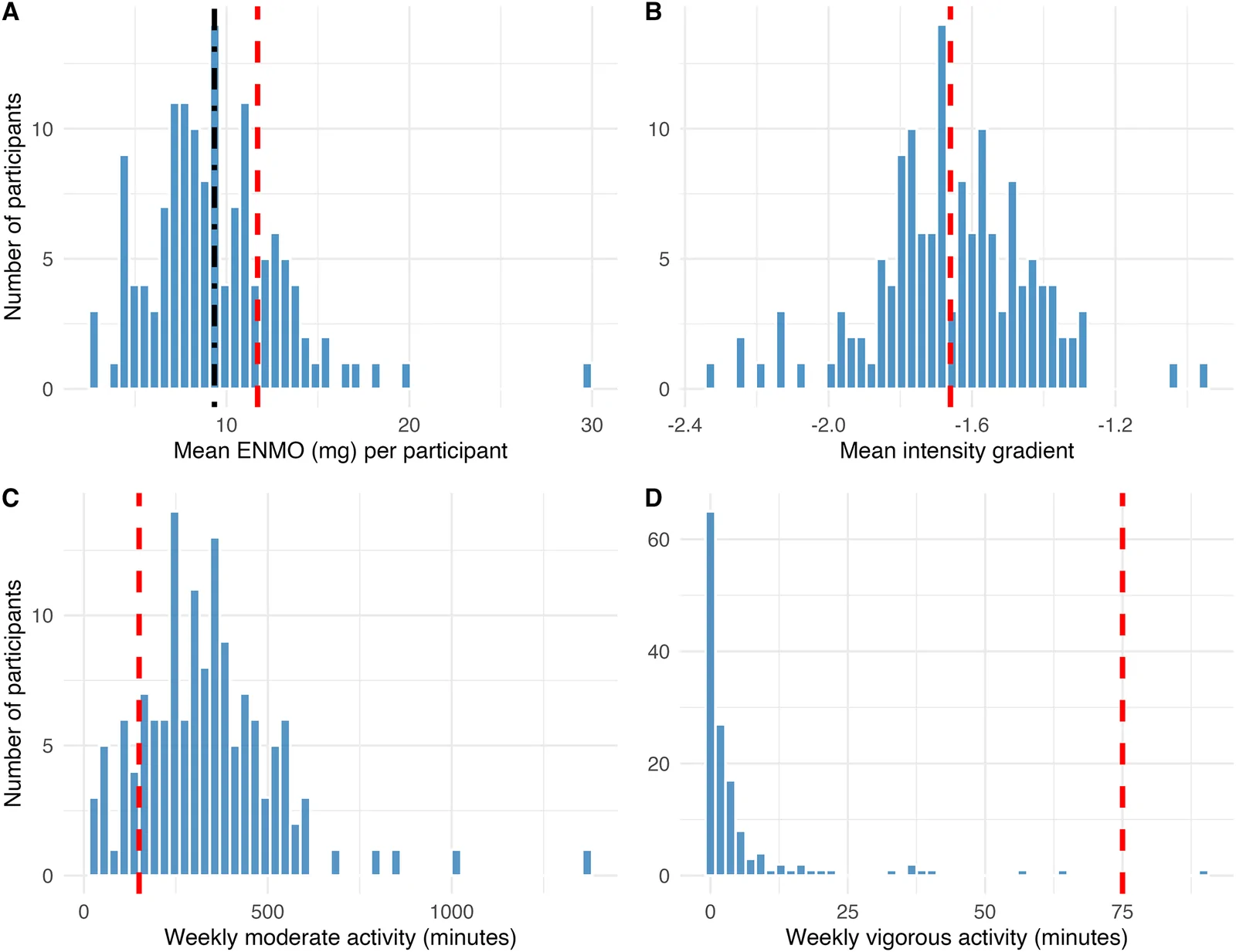
Distribution of accelerometer-derived activity metrics in participants with post-COVID condition (PCC). (A) Mean daily ENMO (mg); black dashed line = PCC cohort mean (9.3 mg), red dashed line = NAKO population mean (11.7 mg). (B) Red dashed line = Mean daily intensity gradient (−1.67). (C) Weekly moderate activity; red dashed line = WHO guideline (≥150 min.week^-1^). (D) Weekly vigorous activity; red dashed line = WHO guideline (≥75 min.week^-1^). All data were obtained from hip-worn ActiGraph devices processed using ENMO thresholds (Hildebrand et al., 2016).

Table 2 summarises key PA parameters of the participants. Participants accumulated a median of 45.9 min/day and 0.16 min/day of moderate and vigorous PA. Most MVPA occurred in short bouts of 1–10 minutes. Overall, 122/141 (86.5%) achieved WHO PA guidelines of at least 150 minutes of moderate intensity activity per week, while only 1/141 (0.7%) achieved at least 75 minutes of vigorous PA (Fig 1C-D).

**Table 2:** Physical activity measures of participants with valid accelerometry data.

Variables	N = 141
Wear time, days	8.0 [7.0–8.0]
Wear time, hours.day^-1^	14.2 [13.2–15.0]
ENMO, mg	9.03 [7.2–11.2]
Intensity gradient	−1.67 [−1.8 – −1.5]
Inactive, min.day^-1^	1048 [996–1110]
LPA, min.day^-1^	25.3 [20.0–32.2]
MPA, min.day-^1^	45.9 [32.1-61.2]
VPA, min.day^-1^	0.16 [0.04–0.6]
30min MVPA bouts, n	0.00 [0.0–0.0]
10min – 30 min MVPA bouts, n	4.8 [0.0–11.3]
1min – 10 min MVPA bouts, n	15 [7.8–22.6]

Data are median [IQR]; mg: milligravity units; LPA: Light physical activity; MPA: Moderate physical activity; MVPA: Moderate-to-vigorous physical activity; VPA: Vigorous physical activity.

The median ENMO was 9.03 mg, and the median intensity gradient was −1.67. The daily mean ENMO of our participants was 9.3 mg, whereas the mean ENMO in the German NAKO population study was 11.7 mg. Overall, 111/141 (78.7%) participants had lower mean ENMO values compared to the NAKO reference population. The mean intensity gradient was −1.66 (Fig 1A-B).

**Fig 2:**
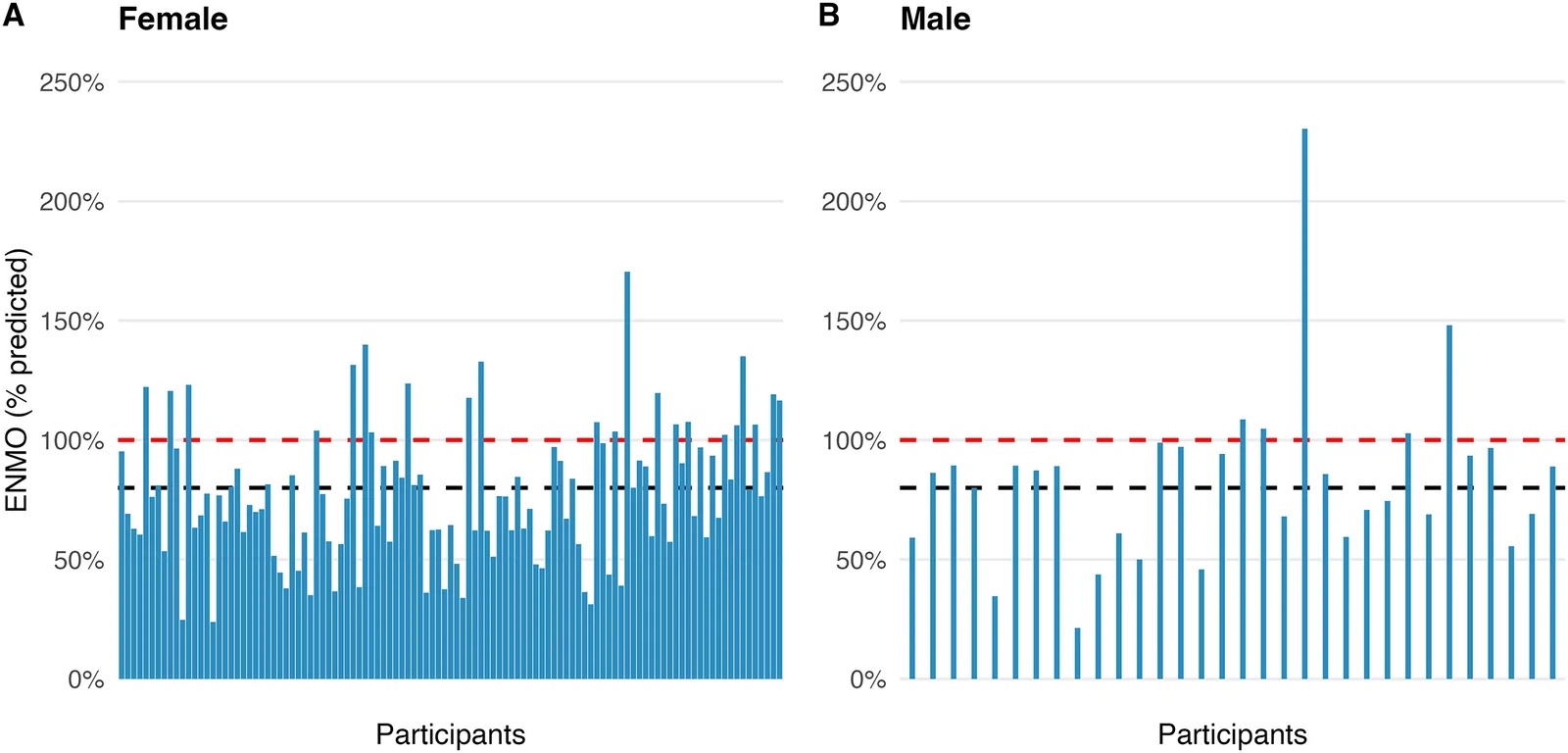
ENMO (mg) values relative to a representative German reference population (NAKO). For each participant, ENMO (mg) is expressed as a percentage of the age- and sex-specific reference from the NAKO study. The red dashed line marks 100% (equal to the reference); the black dashed line marks 80% of the predicted value; bars above/below indicate higher/lower ENMO values than the reference.

Mean ENMO values were highest among men aged 30–39 years and women aged 60–69 years, while the lowest values were observed in men aged 70 years or older and women aged 50–59 years (Table 3). ENMO values were consistently lower across groups and sexes, compared to the German NAKO reference population (Table 3, Fig 2). Fig 2 shows percent predicted ENMO values for each participant, stratified by sex. Among males and females, 84.4% and 79.8% had ENMO values below 100% of the predicted value, respectively, while 43.8% and 56.9% had ENMO values below 80%, respectively (Fig 2).

**Table 3:** Mean ENMO (mg) values among age groups and sex compared to a German reference population (NAKO).

Age, years	Men	NAKO	Percent predicted	Women	NAKO	Percent predicted
<30	11.6 ± 2.5	12.9 ± 4.1	90.2	10.3 ± 4.6	12.4 ± 3.7	82.9
	n = 5	n = 2660		n = 13	n = 3024	
30-39	12.0 ± 7.4	12.9 ± 3.8	93.1	9.0 ± 2.7	12.2 ± 3.5	74.2
	n = 8	n = 3046		n = 19	n = 3498	
40-49	9.7 ± 3.8	12.8 ± 3.9	76.1	8.5 ± 2.8	12.1 ± 3.5	70.6
	n = 10	n = 7841		n = 36	n = 8725	
50-59	10.4 ± 4.2	12.2 ± 4.0	85.3	8.3 ± 3.4	11.6 ± 3.3	71.9
	n = 6	n = 8092		n = 26	n = 9125	
60-69	7.4 ± 1.4	10.5 ± 3.5	70.5	10.5 ± 2.5	10.3 ± 3.1	101.7
	n = 2	n = 7923		n = 13	n = 7990	
≥ 70	4.2	9.7 ± 3.4	43.7	8.8 ± 0.6	9.5 ± 3.0	92.2
	n = 1	n = 695		n = 2	n = 617	

Data are mean ± SD or %; NAKO: German National Cohort. Calculation of percent predicted ENMO values (mg): (measured value/ predicted value) x 100.

**Fig 3:**
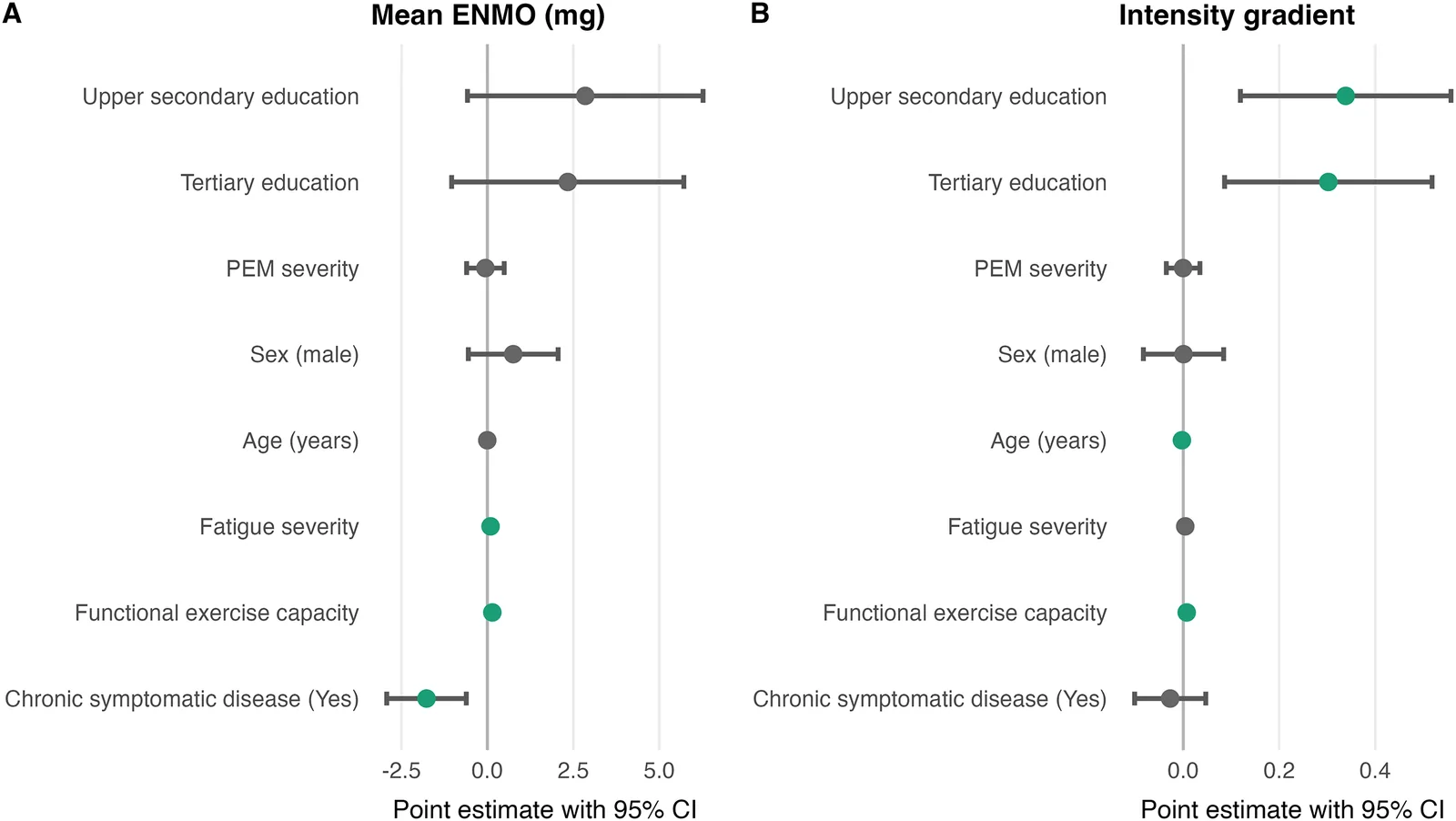
Factors of physical activity (mean ENMO [mg] and intensity gradient) in individuals with PCC. The figure displays adjusted regression coefficients with 95% confidence intervals from the multivariable linear regression model. Higher mean ENMO values indicate higher overall physical activity volume. Fatigue was assessed with the FACIT-Fatigue questionnaire (score 0-52, lower scores indicating more severe fatigue). PEM: Post-exertional malaise.

Table 4 summarises the results of the adjusted multivariable linear regression models for the primary and secondary outcomes, which are visualised in Fig 3. In multivariable linear regression analysis, chronic symptomatic disease other than PCC was negatively associated with mean ENMO (β = –1.77, 95% CI –2.93 to –0.61; p = 0.003). In addition, each additional repetition in the 30-s STS test was associated with higher ENMO values (β = 0.14, 95% CI 0.05 to 0.24; p = 0.003). Higher ENMO values were also associated with less severe fatigue (β = 0.09 per FACIT-Fatigue point, 95% CI 0.02 to 0.16; p = 0.01). Furthermore, each additional repetition in the 30-s STS was associated with a 0.01 higher intensity gradient (95% CI 0.00 to 0.01, p = 0.02). Higher educational levels were also associated with higher (i.e., less negative) intensity gradients, both for tertiary education (β = 0.30, 95% CI 0.09 to 0.52, p = 0.01) and upper secondary education (β = 0.34, 95% CI 0.12 to 0.56, p = 0.003).

**Table 4:** Multivariable linear regression models for primary and secondary outcomes.

Outcomes	n	Estimate (β)	95% CI	p-value
**Mean ENMO**, mg	**141**			
Chronic symptomatic disease		−1.77	−2.93 to −0.61	0.003
Sex, male		0.75	−0.55 to 2.06	0.26
Age, years		−0.00	−0.05 to 0.04	0.89
Functional capacity		0.14	0.05 to 0.24	0.003
Severity of fatigue		0.09	0.02 to 0.16	0.01
Severity of PEM		−0.06	−0.60 to 0.49	0.84
Tertiary education level		2.34	−1.04 to 5.72	0.17
Upper secondary education level		2.85	−0.58 to 6.27	0.10
**Intensity gradient**	**141**			
Chronic symptomatic disease		−0.03	−0.10 to 0.05	0.47
Sex, male		0.00	−0.01 to 0.00	1.00
Age, years		−0.00	−0.01 to 0.00	0.05
Functional capacity		0.01	0.00 to 0.01	0.02
Severity of fatigue		0.00	0.00 to 0.01	0.08
Severity of PEM		−0.00	−0.04 to 0.04	1.00
Tertiary education level		0.30	0.09 to 0.52	0.01
Upper secondary education level		0.34	0.12 to 0.56	0.003

mg: milligravity units; PEM: Post-exertional malaise; missing data: two for 30-s STS, one for Fatigue, one for severity of PEM.

Table S1 in S2 Appendix presents the results of the sensitivity analysis. Mean EQ-VAS was additionally included in the multivariable linear regression models for the primary and secondary outcome. After adjusting for mean EQ-VAS (in addition to all other covariates), presence of chronic symptomatic disease other than PCC remained associated with lower mean ENMO values (β = −1.72, 95% CI −2.86 to −0.58, p = 0.003), and the magnitude was comparable with the primary analysis. In addition, higher functional capacity was associated had higher mean ENMO values (β = 0.12, 95% CI 0.02 to 0.21, p = 0.02), and higher self-reported health status was associated with slightly higher mean ENMO values (β = 0.05, 95% CI 0.00 to 0.09, p = 0.04). For the intensity gradient, higher levels of education (tertiary and secondary) remained significantly associated with a more favourable (i.e., less negative) intensity gradient (β = 0.31, 95% CI 0.10 to 0.53, p = 0.01 and β = 0.35, 95% CI 0.13 to 0.57, p = 0.002, respectively). Functional capacity and age showed marginal associations with intensity gradient (S1 Table in S2 Appendix). No other covariates, including EQ-VAS, showed significant associations with the intensity gradient.

## 4. Discussion

Our findings demonstrate that adults with PCC had overall lower PA levels than the general population, as reflected by a median ENMO of 9.03 mg. Most MVPA was carried out in short bouts of 1–10 minutes rather than in sustained sessions. Chronic symptomatic disease besides PCC and severity of fatigue were associated with lower PA, whereas higher functional capacity was associated with higher PA. For the secondary outcome, the intensity gradient, higher educational attainment and better functional capacity were linked to a more favourable distribution of activity intensities.

Compared to the German NAKO cohort [29], a large and representative sample of the general population, participants in our study had, on average, substantially lower mean ENMO values (9.3 mg vs. 11.7 mg). While existing literature suggests that a 1 mg difference in ENMO is clinically relevant for wrist-worn devices [30], no such minimal clinically important difference has been defined for hip-worn accelerometry. Nonetheless, given that ENMO differences of this magnitude have been associated with meaningful changes in PA and health outcomes in wrist-worn studies, the observed difference likely reflects clinically relevant lower PA levels. This is supported by the large proportion of study participants with ENMO values below their age- and sex-predicted values (i.e., < 100% predicted). A previous national survey reported high levels of PA and sport participation in Switzerland [31], and data from the Federal Office of Public Health showed that 75.7% of the Swiss population met WHO PA recommendations in 2022 [32]. In contrast recent national household survey in Germany suggest lower physical activity levels on a population level [33]. This suggests that the approximately 2.4 mg lower ENMO values in our population compared to the German reference cohort, may even underestimate the difference that would be expected for a Swiss population. Overall, PA was highly heterogeneous across participants, ranging from very low to very high levels. Most recorded activity time fell within the moderate-intensity range, defined as 3–6 metabolic equivalents of task [27]. This is equivalent to brisk walking and routine daily tasks and is supported by the predominance of short 1–10-min activity bouts [34]. Although, a significant proportion of participants achieved ≥150 minutes of moderate activity per week, this value is only the minimum threshold of the current recommendations [10]. In contrast, very little time was accumulated at vigorous intensity. The apparent discrepancy between lower ENMO values and the proportion of participants meeting WHO physical activity recommendations reflects that these metrics capture different dimensions of physical behaviour. ENMO represents overall movement volume, whereas the WHO recommendation is based solely on time accumulated above a moderate-intensity threshold. In our cohort, PA was characterised by minimal vigorous activity and was predominantly accumulated in short, fragmented bouts rather than sustained periods of structured exercise. Consequently, participants could meet the recommended amount of MVPA despite reduced overall movement volume and altered activity patterns. These findings highlight that total activity volume, intensity distribution, bout structure, and vigorous activity provide complementary information that is not captured by weekly MVPA thresholds alone. Accordingly, the WHO MVPA threshold may not fully reflect PA patterns in individuals with post-COVID condition.

While adjusting for relevant co-variates, participants with chronic symptomatic disease exhibited 1.77 mg (95% CI −2.93 to −0.61) lower mean ENMO values compared to those without chronic symptomatic disease, which indicates a clinically relevant difference [30]. Although many chronic conditions are not directly modifiable, mental health status, symptom burden, and body weight are potentially associated with PA, for example, may offer possibilities to enhance habitual PA in individuals with PCC.

Functional capacity and severity of fatigue are further potentially modifiable factors of PA. In our regression analysis, greater functional capacity and less severe fatigue were associated with higher PA levels, suggesting that individuals with better functional reserves tend to be more active in daily life. Although causality cannot be inferred from our data, the findings suggest that regular PA is associated with lower symptom burden (i.e., fatigue) and better functional capacity. However, reverse causation and bidirectional relationships are also possible, as higher symptom burden may reduce PA. This is consistent with evidence from systematic reviews of randomised controlled trials [35] indicating that exercise-based interventions may improve functional capacity and symptoms among individuals with PCC [36].

Furthermore, we found that greater functional capacity and higher educational attainment were associated with a more favourable intensity gradient, indicating a broader distribution of higher-intensity movements throughout the day. This is relevant, as a more favourable intensity gradient has been linked to reduced mortality risk [37]. In the context of PCC, these findings suggest that higher functional capacity or greater health literacy may be associated with a more favourable distribution of activity, despite persistent symptoms. Our findings also align with previous research showing that individuals with higher levels of education are more likely to engage in higher-intensity PA [38]. Together, these results highlight the need to address both physical and educational factors to support more balanced and health-promoting activity patterns in people with PCC.

### 4.1. Strengths and limitations

Strengths of this study include its objectively measured PA avoiding recall and reporting bias inherent in self-reported questionnaires, which are frequently used in studies in this population [39–41]. Raw acceleration data were processed using the open-source R package GGIR, which implements the ENMO metric. This approach offers several advantages over proprietary “black-box” algorithms (e.g., counts.min^-1^), including full transparency and device-independent comparability. ENMO-based metrics allow continuous quantification of activity intensity and have been validated against energy expenditure and health outcomes in diverse populations [42–44]. With mean ENMO (reflecting total activity volume) and the intensity gradient (reflecting the distribution of activity intensities), we provide complementary insights into participants’ physical activity profiles. Finally, the data were collected within a randomised controlled trial, ensuring standardised device use, wear-time instructions, and data collection procedures across all participants, thereby enhancing data quality and internal consistency.

This study has limitations. First, we did not collect sleep data from the accelerometers. Sleep-wake patterns can influence both magnitude and distribution of activity intensity measures such as ENMO and the intensity gradient. The absence of sleep data therefore limits the comparability with studies that include 24-hours activity-rest cycles. We chose not to record sleep with the available hip worn device to minimise participant burden, particularly given that fatigue and PEM are common symptoms in persons with PCC [45].

Second, we used PA reference data from a large population-based German NAKO cohort because no large-scale population-based reference data, including ENMO metrics and covering the age range of our study population, are available for Switzerland. Notably, the same accelerometer device and sensor placement were used in both studies, thereby reducing potential measurement-related heterogeneity. However, differences in PA behaviour between countries, as well as differences in accelerometry protocols (e.g., sampling rates and wear-time validation criteria), may affect the validity of our comparisons and potentially introduce bias. Consequently, comparisons with the NAKO cohort should be considered descriptive rather than a strict benchmark. Third, we expected to observe associations between PA and PEM severity, which was not the case in our study. Although, we captured the presence and severity of PEM using a single question, we did not apply a validated instrument to assess PEM as a multifaceted construct encompassing physical, cognitive, and affective dimensions, with delayed onset and variable duration and intensity [46]. This limitation may reduce the validity of our assessment and partly explain the absence of an observed association. Given that PEM is considered a cardinal symptom of PCC [45] and may substantially influence activity behaviour and tolerance to exertion, our findings regarding physical activity patterns and potentially modifiable correlates should be interpreted with caution. In particular, our cross-sectional data do not permit conclusions regarding the safety or effectiveness of physical reconditioning or activity promotion strategies in individuals experiencing PEM. Finally, our analysis was cross-sectional and based on baseline data of a randomised controlled trial, causal relationships between PA and its factors cannot be inferred.

## 5. Conclusion

In conclusion, these results suggest that, beyond non-modifiable health conditions, potentially modifiable factors including fatigue, functional capacity, and educational attainment are associated with physical activity in individuals with post-COVID 19 condition. These factors may represent potential targets for future interventions aimed at supporting physical activity, although causal relationships cannot be inferred from the present study.

## Supporting information

S1 Appendix Fig S1. Flow diagram of participant inclusion and accelerometer data processing. (DOCX)

S2 Appendix Table S1. Multivariable linear regression models for primary and secondary outcomes with EQ-VAS included as covariate. (DOCX)

S3 Appendix Raw data. (XLSX)
