# Supplementary material for: Physical activity in post-COVID-19 condition: A cross-sectional study
Source: PLoS One. 2026 Sep 1;21(9):e0357465. doi: 10.1371/journal.pone.0357465 (PMC13533376; doi:10.1371/journal.pone.0357465)
Supplement: Supplementary file 2 [file pone.0357465.s002.docx]

**Table S1. Multivariable linear regression models for primary and secondary outcomes with EQ-VAS included as covariate**

| **Outcomes** | | | **n** | **Estimate** | **95% CI** | **p-value** |
| --- | --- | --- | --- | --- | --- | --- |
| **Mean ENMO, mg** | | | **141** |  |  |  |
|  | | Chronic symptomatic disease |  | -1.72 | -2.86 to -0.58 | 0.003 |
|  | | Sex |  | 0.93 | -0.37 to 2.23 | 0.16 |
|  | | Age |  | -0.00 | -0.05 to 0.04 | 0.88 |
|  | | Functional capacity* |  | 0.12 | 0.02 to 0.21 | 0.02 |
|  | | Severity of fatigue |  | 0.05 | -0.02 to 0.13 | 0.18 |
|  | | Severity of PEM |  | 0.22 | -0.38 to 0.82 | 0.47 |
|  | | Tertiary education level |  | 2.64 | -0.71 to 5.98 | 0.12 |
|  | | Upper secondary education level |  | 3.11 | -0.28 to 6.50 | 0.07 |
|  | | Mean EQ-VAS |  | 0.05 | 0.00 to 0.09 | 0.04 |
| **Intensity gradient** | | | **141** |  |  |  |
|  | Chronic symptomatic disease | | | -0.03 | -0.10 to 0.05 | 0.50 |
|  | Sex | | | 0.01 | -0.08 to 0.09 | 0.88 |
|  | Age | | | -0.00 | -0.01 to 0.00 | 0.05 |
|  | Functional capacity* | | | 0.01 | 0.00 to 0.01 | 0.05 |
|  | Severity of fatigue | | | 0.00 | -0.00 to 0.01 | 0.28 |
|  | Severity of PEM | | | 0.01 | -0.03 to 0.05 | 0.65 |
|  | Tertiary education level | | | 0.31 | 0.10 to 0.53 | 0.01 |
|  | Secondary education level | | | 0.35 | 0.13 to 0.57 | 0.002 |
|  | Mean EQ-VAS | | | 0.00 | -0.00 to 0.00 | 0.27 |

mg: milligravity units; PEM: Post-exertional malaise; EQ-VAS: EuroQol Visual Analogue Scale. Missing data: two for 30s STS, one for Fatigue, one for severity of PEM.

*Functional capacity was assessed with the 30second sit-to-stand test (30s STS).
