# Supplementary material for: Physical activity in post-COVID-19 condition: A cross-sectional study
Source: PLoS One. 2026 Sep 1;21(9):e0357465. doi: 10.1371/journal.pone.0357465 (PMC13533376; doi:10.1371/journal.pone.0357465)
Supplement: Supplementary file 4 [file pone.0357465.s006.docx]

**Response to the Editor and Reviewer**

**Manuscript:** PONE-D-25-64893

**Title:** Physical activity in post-COVID-19 condition: a cross-sectional study

**Response to the editor**

We thank the reviewer and the editor for their careful evaluation of our manuscript entitled « Physical activity in post-COVID-19 condition: a cross-sectional study».

We provide point-by-point responses to the reviewers' questions and hope that we have addressed all points to their satisfaction.

**Reviewer #1:** Thank you for addressing all of my concerns.

Response: Thank you again for the valuable feedback.

**Reviewer #2:** The manuscript addresses a relevant topic and provides useful objective data on physical activity in adults with post-COVID-19 condition. The use of accelerometry, raw acceleration metrics, ENMO, and the intensity gradient is a clear strength, particularly in a field where many studies rely on self-reported physical activity. The study is generally well designed, the methods are adequately described, and the data support the main conclusions.

I consider the manuscript suitable for publication after minor revision. I suggest the authors address the following points.

Response: We thank the reviewer for their careful review and valuable feedback.

The interpretation should remain cautious because the analysis is cross-sectional. Some statements in the Discussion and Conclusion imply that improving fatigue, functional capacity, or education-related factors may increase physical activity. This is plausible, but the present design only supports associations. I recommend softening the causal and intervention-oriented wording.

Response: We have carefully revised the Results, Discussion, and Conclusion sections to ensure that all statements are framed in terms of associations and that causal or intervention-oriented wording has been removed or appropriately softened.

We agree that the Conclusions section was somewhat misleading and speculative and included statements that were not fully supported by the data. We have therefore revised this section accordingly.

Page 19: “In conclusion, these results suggest that, beyond non-modifiable health conditions, potentially modifiable factors including fatigue, functional capacity, and educational attainment are associated with physical activity in individuals with post-COVID 19 condition. These factors may represent potential targets for future interventions aimed at supporting physical activity, although causal relationships cannot be inferred from the present study”.

The comparison with the NAKO reference population is useful, but it should be framed clearly as an external descriptive comparison rather than as evidence from a matched control group. Potential differences in recruitment, country, sample characteristics, device placement, and context should be acknowledged more explicitly.

Response: Thank you for this important comment. We added the following information to the Statistical Analysis and Limitations sections:

Page 8: “Of note, the NAKO reference population was used solely for external descriptive comparisons with our study population and cannot be considered a matched control group”.

Pages 18/19: “Second, we used PA reference data from a large population-based German NAKO cohort because no large-scale population-based reference data, including ENMO metrics and covering the age range of our study population, are available for Switzerland. Notably, the same accelerometer device and sensor placement were used in both studies, thereby reducing potential measurement-related heterogeneity. However, differences in PA behavior between countries, as well as differences in accelerometry protocols (e.g., sampling rates and wear-time validation criteria), may affect the validity of our comparisons and potentially introduce bias. Consequently, comparisons with the NAKO cohort should be considered descriptive rather than a strict benchmark.”

The manuscript reports low overall ENMO, while most participants met the WHO recommendation for moderate physical activity. This apparent discrepancy should be explained more clearly. The authors could emphasize that total activity volume, intensity distribution, bout structure, and vigorous activity provide information that is not fully captured by weekly moderate activity thresholds.

Response: The reviewer is correct. Although most participants accumulated ≥150 min/week of MVPA, ENMO values remained lower than those observed in the reference population. This apparent discrepancy likely reflects the fact that these metrics capture different dimensions of physical activity behavior. In our cohort, physical activity was characterized by very little vigorous activity and was predominantly accumulated in short, fragmented bouts rather than in sustained periods of structured exercise. Consequently, participants could achieve the recommended amount of MVPA despite exhibiting reduced overall movement volume and altered activity patterns. These findings suggest that the WHO MVPA threshold alone may not adequately capture impairments in physical activity behavior in individuals with post-COVID condition.

We added the following paragraph to the Discussion section (page 16/17):

“The apparent discrepancy between lower ENMO values and the proportion of participants meeting WHO physical activity recommendations reflects that these metrics capture different dimensions of physical behaviour. ENMO represents overall movement volume, whereas the WHO recommendation is based solely on time accumulated above a moderate-intensity threshold. In our cohort, PA was characterized by minimal vigorous activity and was predominantly accumulated in short, fragmented bouts rather than sustained periods of structured exercise. Consequently, participants could meet the recommended amount of MVPA despite reduced overall movement volume and altered activity patterns. These findings highlight that total activity volume, intensity distribution, bout structure, and vigorous activity provide complementary information that is not captured by weekly MVPA thresholds alone. Accordingly, the WHO MVPA threshold may not fully reflect PA patterns in individuals with post-COVID condition.“

The assessment of post-exertional malaise is a limitation. PEM is central to post-COVID-19 condition, but it was assessed with a single item rather than a validated multidimensional instrument. This limitation should be given more weight, especially when discussing physical reconditioning or activity promotion.

Response: We agree that the assessment of PEM is a major limitation of our study. To be transparent about this limitation, we already acknowledged it in the Methods section and have further emphasised it in the Discussion.

Page 8: “We did not assess PEM using a validated instrument, which limits the validity of our evaluation of this complex symptom.”

Further, we extended the paragraph in the Limitations section to give this limitation more weight, as suggested.

Page 19: “Third, we expected to observe associations between PA and PEM severity, which was not the case in our study. Although, we captured the presence and severity of PEM using a single question, we did not apply a validated instrument to assess PEM as a multifaceted construct encompassing physical, cognitive, and affective dimensions, with delayed onset and variable duration and intensity [46]. This limitation may reduce the validity of our assessment and partly explain the absence of an observed association. Given that PEM is considered a cardinal symptom of PCC [45] and may substantially influence activity behaviour and tolerance to exertion, our findings regarding physical activity patterns and potentially modifiable correlates should be interpreted with caution. In particular, our cross-sectional data do not permit conclusions regarding the safety or effectiveness of physical reconditioning or activity promotion strategies in individuals experiencing PEM”.

Please correct minor language and typographical issues. For example, “accelerometery” should be “accelerometry”, “modelsfor” should be separated, “literature suggest” should be “literature suggests”, and “age- and se-specific” appears to be a typographical error.

Response: Done, thank you.

Overall, this is a valuable and technically sound study. With these minor revisions, the manuscript would provide a clear and useful contribution to the literature on physical activity in post-COVID-19 condition.

Response: Thank you.
