# Supplementary material for: Physical activity in post-COVID-19 condition: A cross-sectional study
Source: PLoS One. 2026 Sep 1;21(9):e0357465. doi: 10.1371/journal.pone.0357465 (PMC13533376; doi:10.1371/journal.pone.0357465)
Supplement: Supplementary file 5 [file pone.0357465.s001.docx]

Fig S1. Flow diagram of participant inclusion and accelerometer data processing
