# Supplementary material for: Physical activity in post-COVID-19 condition: A cross-sectional study
Source: PLoS One. 2026 Sep 1;21(9):e0357465. doi: 10.1371/journal.pone.0357465 (PMC13533376; doi:10.1371/journal.pone.0357465)
Supplement: Supplementary file 6 [file pone.0357465.s005.docx]

**Response to the Editor and Reviewer**

**Manuscript:** PONE-D-25-64893

**Title:** Physical activity in post-COVID-19 condition: a cross-sectional study

**Response to the editor**

We thank the reviewer and the editor for their careful evaluation of our manuscript entitled « Physical activity in post-COVID-19 condition: a cross-sectional study».

The manuscript has undergone substantial revision incorporating all reviewer comments. We provide point-by-point responses to the reviewers' questions and hope that we have addressed all points to their satisfaction.

**Figures:** We have uploaded higher-quality versions of Figures 1–3.

**Raw data:** We removed an empty column from the Excel spreadsheet and uploaded a revised version.

**Funding statement:** We have double-checked the funding statement to ensure that the *Funding Information* and *Financial Disclosure* sections are consistent.

**Supporting Information:** We apologize for the omission of the Supporting Information file (*S1 Supporting Information*). We have uploaded the flow chart together with the revised manuscript.

**Reviewers' comments:**

Reviewer's Responses to Questions

**Comments to the Author**

Reviewer #1: Summary: This paper examines accelerometer-derived physical activity levels among patients with post-COVID-19 condition. This is an important topic, and the study uses appropriately rigorous methodology. I have some minor comments regarding the methodology and conclusions which are outlined below.

Response: We thank the reviewer for their careful review and for acknowledging the rigorous research methodology.

Detailed Comments:

Line 168-169: Please provide more details regarding the GGIR non-wear algorithm for those who are not familiar with this approach. E.g. is it a certain period of zero counts?

Response: Non-wear time was detected using the GGIR package (version 3.2.6) validated raw-acceleration algorithm. The algorithm evaluates the variability of acceleration in each axis across consecutive moving windows and classifies periods as non-wear when both the standard deviation and the acceleration range remain below predefined thresholds, consistent with the device being stationary. Thus, non-wear detection is based on sustained low acceleration variability rather than a predefined period of zero counts. This approach differs from traditional count-based methods, which typically define non-wear as a prolonged period of zero counts.

We added the following paragraph to the manuscript (page 7): *“Non-wear periods were identified and imputed according to GGIR’s standard algorithms based on sustained inactivity. Non-wear periods were identified using GGIR’s raw-acceleration algorithm rather than a zero-count rule. The algorithm evaluates the standard deviation and value range of acceleration in each axis across 60-minute moving windows with 15-minute increments and classifies periods as non-wear when these low-variability criteria are met for at least two of the three axes, consistent with the device being stationary rather than worn”.*

Line 170-171: How was waking and sleep time identified? Was it self-reported by participants or identified via the GGIR algorithm?

Response: Waking and sleep time were not directly measured by the accelerometer. During data processing with GGIR, these overnight periods were identified as non-wear time. We chose this approach to minimize participant burden and avoid potential sleep disturbance from wearing a hip-mounted accelerometer overnight, particularly given the high prevalence of sleep problems among individuals with post-COVID conditions (see LIMITATIONS section).

Line 172: Can you give an example of 90% adherence? E.g. is it considered a “bout” of PA if 9 out of 10 minutes are vigorous PA?

Response: Yes, that’s correct. We used the default GGIR settings, in which activity bouts are defined using a 90% adherence criterion. This means that at least 90% of the epochs within the specified bout duration must meet or exceed the relevant intensity threshold. For example, in a 10-minute bout, up to 10% of the epochs (approximately 1 minute if using 1-minute epochs) may fall below the threshold, while the remaining 90% must satisfy the threshold. Thus, a 10-minute period with 9 minutes above the threshold and 1 minute below would still be classified as a valid bout.

We added the following sentences to the manuscript (pages 8-9):

*“PA intensities were classified using ENMO thresholds of 47.4 mg for light, 69.1 mg for moderate, and 258.7 mg for vigorous PA, with activity bouts defined using a 90% adherence criterion [26,27], meaning that at least 90% of epochs within a bout were required to meet the corresponding intensity threshold (e.g., in a 10-minute bout, up to one minute could fall below the threshold).”*

Line 176: Can you explain what “parallel mode” means?

Response: Parallel processing in GGIR refers to the use of multiple CPU cores to analyse individual accelerometer files simultaneously rather than sequentially. This computational approach is used to reduce processing time in large datasets. Importantly, parallelization does not alter any analytical steps; calibration, ENMO computation, and non-wear detection are identical to single-core processing, with only the speed of execution being affected.

We added the following paragraph to the manuscript (page 8):

*”Activity recordings with a minimum wear time of 10 hours per day with at least three valid weekdays and one valid weekend day were included in the analysis. All analyses were performed using parallel processing to reduce computation time in this large dataset. This refers to the simultaneous processing of multiple files across available CPU cores and does not affect the underlying analytical procedures. Default GGIR calibration procedures were applied, and non-wear time was identified using the built-in raw acceleration–based algorithm”.*

Line 183: More details are needed regarding how the predicted values were calculated, as this is a core part of the results, but few details are provided on how this was actually calculated.

Response: Thank you for this comment. Percent predicted values were calculated as the participant's observed mean ENMO divided by the age- and sex-specific reference value from the NAKO cohort, multiplied by 100. We agree that this was not sufficiently explicit in the Methods section and have added a clarifying sentence to the manuscript.

The revised sentence reads (page 8):

*“In addition, we calculated percent predicted values by dividing each participant’s observed mean ENMO value by the corresponding age- and se-specific reference value derived from a large population-based German cohort (NAKO) [29], and multiplying the result by 100.”*

In addition, we provide an example calculation for the reviewer. NAKO predicted values are reported in Table 3.

**Example calculation for a women aged 38 years.**

Predicted ENMO value (NAKO): 12.2 mg

Measured ENMO value: 9.9 mg

Percent predicted = (Measured value / Predicted value) * 100 = (9.9 / 12.2) * 100 = **81.14**

We added the following information to the legend of Table 3:

*“Calculation of percent predicted ENMO values (mg): (measured value / predicted value) x 100”.*

Line 221: Why was 80% of the predicted value use as a key outcome, rather than 100% of the predicted value? It is hard to interpret what it means that only 43.8% of males meet 80% of predicted values.

Response: Thank you for this suggestion. We have added the proportion of participants below 100% predicted. We agree that 100% predicted represents the age- and sex-specific mean reference value from the NAKO cohort and therefore provides an additional descriptive comparison with the reference population. As the published reference data do not provide percentile distributions or prediction equations, we did not derive a lower limit of normal.

The 80% predicted threshold was retained as a pragmatic benchmark commonly used in clinical practice and is presented for descriptive purposes only.

Line 345: I disagree with the statement that a “large proportion” of participants were below 80% of predicted – it was < 50% of males and just over 50% of females. It would be more intuitive to know what % is less than 100% of predicted, but it is also hard to interpret without details on how the predictions were actually calculated.

Response: We thank the reviewer for the valuable suggestion. In the revised manuscript, we primarily focus on 100% of the predicted value. In the RESULTS section, we provide the percentage of males and females below 100% of the predicted value. The results indicate that a large proportion of our study sample is below the predicted value from the NAKO reference population.

“Among males and females, 84.4% and 79.8% had ENMO values below 100% predicted, respectively”.

Line 366-373: It seems equally likely that higher symptom burden may lead to lower PA, or there may be a bi-directional relationship. I would suggest acknowledging all of these possibilities.

Response: This is a very good point. We changed the sentence to address possible bi-directional relationships. It reads (page 17):

*“Although causality cannot be inferred from our data, the findings suggest that regular PA is associated with lower symptom burden (i.e., fatigue) and better functional capacity. However, reverse causation and bidirectional relationships are also possible, as higher symptom burden may reduce PA.”*
