# Supplementary material for: Physical activity in post-COVID-19 condition: A cross-sectional study
Source: PLoS One. 2026 Sep 1;21(9):e0357465. doi: 10.1371/journal.pone.0357465 (PMC13533376; doi:10.1371/journal.pone.0357465)
Supplement: Supplementary file 7 [file pone.0357465.s007.docx]

**Response to Editor**

Thank you for your thoughtful responses to the previous editor and reviewer's comments.  One minor comment regarding Figure S1 in the Supporting Information: this figure does not appear to be referenced in the current manuscript.

Response: We have cite Figure S1 on page 9.
