# Supplementary material for: Physical activity in post-COVID-19 condition: A cross-sectional study
Source: PLoS One. 2026 Sep 1;21(9):e0357465. doi: 10.1371/journal.pone.0357465 (PMC13533376; doi:10.1371/journal.pone.0357465)
Supplement: Peer review history

# Peer review: Decision Letter 0

**DOI:** 10.1371/journal.pone.0357465.r001

**Authors:** Xin Sun

28 May 2026

Dear Dr. Radtke,

Thank you for submitting your manuscript to PLOS ONE. After careful consideration, we feel that it has merit but does not fully meet PLOS ONE’s publication criteria as it currently stands. Therefore, we invite you to submit a revised version of the manuscript that addresses the points raised during the review process.

Your manuscript has been evaluated by one reviewer. This reviewer has listed several scientific concerns. Please read carefully and address them one by one in your revision.

Please submit your revised manuscript by Jul 12 2026 11:59PM. If you will need more time than this to complete your revisions, please reply to this message or contact the journal office at plosone@plos.org. When you're ready to submit your revision, log on to https://www.editorialmanager.com/pone/ and select the 'Submissions Needing Revision' folder to locate your manuscript file.

- A letter that responds to each point raised by the academic editor and reviewer(s). You should upload this letter as a separate file labeled 'Response to Reviewers'.
- A marked-up copy of your manuscript that highlights changes made to the original version. You should upload this as a separate file labeled 'Revised Manuscript with Track Changes'.
- An unmarked version of your revised paper without tracked changes. You should upload this as a separate file labeled 'Manuscript'.

If you would like to make changes to your financial disclosure, please include your updated statement in your cover letter. Guidelines for resubmitting your figure files are available below the reviewer comments at the end of this letter.

If applicable, we recommend that you deposit your laboratory protocols in protocols.io to enhance the reproducibility of your results. Protocols.io assigns your protocol its own identifier (DOI) so that it can be cited independently in the future. For instructions see: https://journals.plos.org/plosone/s/submission-guidelines#loc-laboratory-protocols. Additionally, PLOS ONE offers an option for publishing peer-reviewed Lab Protocol articles, which describe protocols hosted on protocols.io. Read more information on sharing protocols at https://plos.org/protocols?utm_medium=editorial-email&utm_source=authorletters&utm_campaign=protocols.

As the corresponding author, your ORCID iD is verified in the submission system and will appear in the published article. PLOS supports the use of ORCID, and we encourage all coauthors to register for an ORCID iD and use it as well. Please encourage your coauthors to verify their ORCID iD within the submission system before final acceptance, as unverified ORCID iDs will not appear in the published article. *Only*  the individual author can complete the verification step; PLOS staff *cannot*  verify ORCID iDs on behalf of authors.

We look forward to receiving your revised manuscript.

Kind regards,

Xin Sun, PhD

Staff Editor

PLOS One

Journal Requirements:

When submitting your revision, we need you to address these additional requirements.

1. Please ensure that your manuscript meets PLOS ONE's style requirements, including those for file naming. The PLOS ONE style templates can be found at

https://journals.plos.org/plosone/s/file?id=wjVg/PLOSOne_formatting_sample_main_body.pdf and

https://journals.plos.org/plosone/s/file?id=ba62/PLOSOne_formatting_sample_title_authors_affiliations.pdf

2. We note that the grant information you provided in the ‘Funding Information’ and ‘Financial Disclosure’ sections do not match.

When you resubmit, please ensure that you provide the correct grant numbers for the awards you received for your study in the ‘Funding Information’ section.

3. We notice that the figure is missing in your Supporting Information file [S1_Supporting Information]. Please kindly revise as necessary and re-upload.

4. If the reviewer comments include a recommendation to cite specific previously published works, please review and evaluate these publications to determine whether they are relevant and should be cited. There is no requirement to cite these works unless the editor has indicated otherwise.

[Note: HTML markup is below. Please do not edit.]

Reviewers' comments:

Reviewer's Responses to Questions

**Comments to the Author**

1. Is the manuscript technically sound, and do the data support the conclusions?

Reviewer #1: Partly

**********

2. Has the statistical analysis been performed appropriately and rigorously?

Reviewer #1: Yes

**********

3. Have the authors made all data underlying the findings in their manuscript fully available?

The PLOS Data policy

Reviewer #1: Yes

**********

4. Is the manuscript presented in an intelligible fashion and written in standard English?

Reviewer #1: Yes

**********

Reviewer #1: Summary: This paper examines accelerometer-derived physical activity levels among patients with post-COVID-19 condition. This is an important topic, and the study uses appropriately rigorous methodology. I have some minor comments regarding the methodology and conclusions which are outlined below.

Detailed Comments:

Line 168-169: Please provide more details regarding the GGIR non-wear algorithm for those who are not familiar with this approach. E.g. is it a certain period of zero counts?

Line 170-171: How was waking and sleep time identified? Was it self-reported by participants or identified via the GGIR algorithm?

Line 172: Can you give an example of 90% adherence? E.g. is it considered a “bout” of PA if 9 out of 10 minutes are vigorous PA?

Line 176: Can you explain what “parallel mode” means?

Line 183: More details are needed regarding how the predicted values were calculated, as this is a core part of the results, but few details are provided on how this was actually calculated.

Line 221: Why was 80% of the predicted value use as a key outcome, rather than 100% of the predicted value? It is hard to interpret what it means that only 43.8% of males meet 80% of predicted values.

Line 345: I disagree with the statement that a “large proportion” of participants were below 80% of predicted – it was < 50% of males and just over 50% of females. It would be more intuitive to know what % is less than 100% of predicted, but it is also hard to interpret without details on how the predictions were actually calculated.

Line 366-373: It seems equally likely that higher symptom burden may lead to lower PA, or there may be a bi-directional relationship. I would suggest acknowledging all of these possibilities.

Line 413-416: These conclusions RE mental health and weight loss do not seem to tie to the key results of this study, aside from a brief mention in lines 363-365. I strongly recommend re-wording the conclusion to focus on the key findings of the paper.

**********

what does this mean?). If published, this will include your full peer review and any attached files.

If you choose “no”, your identity will remain anonymous but your review may still be made public.

**Do you want your identity to be public for this peer review?**  For information about this choice, including consent withdrawal, please see our Privacy Policy

Reviewer #1: No

**********

[NOTE: If reviewer comments were submitted as an attachment file, they will be attached to this email and accessible via the submission site. Please log into your account, locate the manuscript record, and check for the action link "View Attachments". If this link does not appear, there are no attachment files.]

To ensure your figures meet our technical requirements, please review our figure guidelines: https://journals.plos.org/plosone/s/figures

You may also use PLOS’s free figure tool, NAAS, to help you prepare publication quality figures: https://journals.plos.org/plosone/s/figures#loc-tools-for-figure-preparation.

NAAS will assess whether your figures meet our technical requirements by comparing each figure against our figure specifications.

# Author comment: Author response to Decision Letter 1

**DOI:** 10.1371/journal.pone.0357465.r002

8 Jun 2026

Please see uploaded document entitled "Response to Reviewers"

Attachment Submitted filename: Response to Reviewers.docx

# Peer review: Decision Letter 1

**DOI:** 10.1371/journal.pone.0357465.r003

**Authors:** Mukesh Sinha

26 Jun 2026

Dear Dr. Radtke,

Thank you for submitting your manuscript to PLOS ONE. After careful consideration, we feel that it has merit but does not fully meet PLOS ONE’s publication criteria as it currently stands. Therefore, we invite you to submit a revised version of the manuscript that addresses the points raised during the review process.

Please submit your revised manuscript by Aug 10 2026 11:59PM. If you will need more time than this to complete your revisions, please reply to this message or contact the journal office at plosone@plos.org. When you're ready to submit your revision, log on to https://www.editorialmanager.com/pone/ and select the 'Submissions Needing Revision' folder to locate your manuscript file.

- A letter that responds to each point raised by the academic editor and reviewer(s). You should upload this letter as a separate file labeled 'Response to Reviewers'.
- A marked-up copy of your manuscript that highlights changes made to the original version. You should upload this as a separate file labeled 'Revised Manuscript with Track Changes'.
- An unmarked version of your revised paper without tracked changes. You should upload this as a separate file labeled 'Manuscript'.

If you would like to make changes to your financial disclosure, please include your updated statement in your cover letter. Guidelines for resubmitting your figure files are available below the reviewer comments at the end of this letter.

If applicable, we recommend that you deposit your laboratory protocols in protocols.io to enhance the reproducibility of your results. Protocols.io assigns your protocol its own identifier (DOI) so that it can be cited independently in the future. For instructions see: https://journals.plos.org/plosone/s/submission-guidelines#loc-laboratory-protocols. Additionally, PLOS ONE offers an option for publishing peer-reviewed Lab Protocol articles, which describe protocols hosted on protocols.io. Read more information on sharing protocols at https://plos.org/protocols?utm_medium=editorial-email&utm_source=authorletters&utm_campaign=protocols.

As the corresponding author, your ORCID iD is verified in the submission system and will appear in the published article. PLOS supports the use of ORCID, and we encourage all coauthors to register for an ORCID iD and use it as well. Please encourage your coauthors to verify their ORCID iD within the submission system before final acceptance, as unverified ORCID iDs will not appear in the published article. *Only*  the individual author can complete the verification step; PLOS staff *cannot*  verify ORCID iDs on behalf of authors.

We look forward to receiving your revised manuscript.

Kind regards,

Mukesh Kumar Sinha, PhD

Academic Editor

PLOS One

**Journal Requirements:**

1. If the reviewer comments include a recommendation to cite specific previously published works, please review and evaluate these publications to determine whether they are relevant and should be cited. There is no requirement to cite these works unless the editor has indicated otherwise.

2. Please review your reference list to ensure that it is complete and correct. If you have cited papers that have been retracted, please include the rationale for doing so in the manuscript text, or remove these references and replace them with relevant current references. Any changes to the reference list should be mentioned in the rebuttal letter that accompanies your revised manuscript. If you need to cite a retracted article, indicate the article’s retracted status in the References list and also include a citation and full reference for the retraction notice.

**Additional Editor Comments:**

overall manuscript is well written, however few concern raised by reviewer need to address prior to acceptance.

Thank you

[Note: HTML markup is below. Please do not edit.]

Reviewers' comments:

Reviewer's Responses to Questions

**Comments to the Author**

Reviewer #1: All comments have been addressed

Reviewer #2: (No Response)

**********

2. Is the manuscript technically sound, and do the data support the conclusions?

Reviewer #1: Yes

Reviewer #2: Yes

**********

3. Has the statistical analysis been performed appropriately and rigorously?

Reviewer #1: Yes

Reviewer #2: Yes

**********

4. Have the authors made all data underlying the findings in their manuscript fully available?

The PLOS Data policy

Reviewer #1: Yes

Reviewer #2: Yes

**********

5. Is the manuscript presented in an intelligible fashion and written in standard English?

Reviewer #1: Yes

Reviewer #2: Yes

**********

Reviewer #1: Thank you for addressing all of my concerns.

Reviewer #2: The manuscript addresses a relevant topic and provides useful objective data on physical activity in adults with post-COVID-19 condition. The use of accelerometry, raw acceleration metrics, ENMO, and the intensity gradient is a clear strength, particularly in a field where many studies rely on self-reported physical activity. The study is generally well designed, the methods are adequately described, and the data support the main conclusions.

I consider the manuscript suitable for publication after minor revision. I suggest the authors address the following points.

The interpretation should remain cautious because the analysis is cross-sectional. Some statements in the Discussion and Conclusion imply that improving fatigue, functional capacity, or education-related factors may increase physical activity. This is plausible, but the present design only supports associations. I recommend softening the causal and intervention-oriented wording.

The comparison with the NAKO reference population is useful, but it should be framed clearly as an external descriptive comparison rather than as evidence from a matched control group. Potential differences in recruitment, country, sample characteristics, device placement, and context should be acknowledged more explicitly.

The manuscript reports low overall ENMO, while most participants met the WHO recommendation for moderate physical activity. This apparent discrepancy should be explained more clearly. The authors could emphasize that total activity volume, intensity distribution, bout structure, and vigorous activity provide information that is not fully captured by weekly moderate activity thresholds.

The assessment of post-exertional malaise is a limitation. PEM is central to post-COVID-19 condition, but it was assessed with a single item rather than a validated multidimensional instrument. This limitation should be given more weight, especially when discussing physical reconditioning or activity promotion.

Please correct minor language and typographical issues. For example, “accelerometery” should be “accelerometry”, “modelsfor” should be separated, “literature suggest” should be “literature suggests”, and “age- and se-specific” appears to be a typographical error.

Overall, this is a valuable and technically sound study. With these minor revisions, the manuscript would provide a clear and useful contribution to the literature on physical activity in post-COVID-19 condition.

**********

what does this mean?). If published, this will include your full peer review and any attached files.

If you choose “no”, your identity will remain anonymous but your review may still be made public.

**Do you want your identity to be public for this peer review?**  For information about this choice, including consent withdrawal, please see our Privacy Policy

Reviewer #1: No

Reviewer #2: No

**********

[NOTE: If reviewer comments were submitted as an attachment file, they will be attached to this email and accessible via the submission site. Please log into your account, locate the manuscript record, and check for the action link "View Attachments". If this link does not appear, there are no attachment files.]

To ensure your figures meet our technical requirements, please review our figure guidelines: https://journals.plos.org/plosone/s/figures

You may also use PLOS’s free figure tool, NAAS, to help you prepare publication quality figures: https://journals.plos.org/plosone/s/figures#loc-tools-for-figure-preparation.

NAAS will assess whether your figures meet our technical requirements by comparing each figure against our figure specifications.

# Author comment: Author response to Decision Letter 2

**DOI:** 10.1371/journal.pone.0357465.r004

29 Jun 2026

Response to the Editor and Reviewer

Manuscript: PONE-D-25-64893

Title: Physical activity in post-COVID-19 condition: a cross-sectional study

Response to the editor

We thank the reviewer and the editor for their careful evaluation of our manuscript entitled « Physical activity in post-COVID-19 condition: a cross-sectional study».

We provide point-by-point responses to the reviewers' questions and hope that we have addressed all points to their satisfaction.

Reviewer #1: Thank you for addressing all of my concerns.

Response: Thank you again for the valuable feedback.

Reviewer #2: The manuscript addresses a relevant topic and provides useful objective data on physical activity in adults with post-COVID-19 condition. The use of accelerometry, raw acceleration metrics, ENMO, and the intensity gradient is a clear strength, particularly in a field where many studies rely on self-reported physical activity. The study is generally well designed, the methods are adequately described, and the data support the main conclusions.

I consider the manuscript suitable for publication after minor revision. I suggest the authors address the following points.

Response: We thank the reviewer for their careful review and valuable feedback.

The interpretation should remain cautious because the analysis is cross-sectional. Some statements in the Discussion and Conclusion imply that improving fatigue, functional capacity, or education-related factors may increase physical activity. This is plausible, but the present design only supports associations. I recommend softening the causal and intervention-oriented wording.

Response: We have carefully revised the Results, Discussion, and Conclusion sections to ensure that all statements are framed in terms of associations and that causal or intervention-oriented wording has been removed or appropriately softened.

We agree that the Conclusions section was somewhat misleading and speculative and included statements that were not fully supported by the data. We have therefore revised this section accordingly.

Page 19: “In conclusion, these results suggest that, beyond non-modifiable health conditions, potentially modifiable factors including fatigue, functional capacity, and educational attainment are associated with physical activity in individuals with post-COVID 19 condition. These factors may represent potential targets for future interventions aimed at supporting physical activity, although causal relationships cannot be inferred from the present study”.

The comparison with the NAKO reference population is useful, but it should be framed clearly as an external descriptive comparison rather than as evidence from a matched control group. Potential differences in recruitment, country, sample characteristics, device placement, and context should be acknowledged more explicitly.

Response: Thank you for this important comment. We added the following information to the Statistical Analysis and Limitations sections:

Page 8: “Of note, the NAKO reference population was used solely for external descriptive comparisons with our study population and cannot be considered a matched control group”.

Pages 18/19: “Second, we used PA reference data from a large population-based German NAKO cohort because no large-scale population-based reference data, including ENMO metrics and covering the age range of our study population, are available for Switzerland. Notably, the same accelerometer device and sensor placement were used in both studies, thereby reducing potential measurement-related heterogeneity. However, differences in PA behavior between countries, as well as differences in accelerometry protocols (e.g., sampling rates and wear-time validation criteria), may affect the validity of our comparisons and potentially introduce bias. Consequently, comparisons with the NAKO cohort should be considered descriptive rather than a strict benchmark.”

The manuscript reports low overall ENMO, while most participants met the WHO recommendation for moderate physical activity. This apparent discrepancy should be explained more clearly. The authors could emphasize that total activity volume, intensity distribution, bout structure, and vigorous activity provide information that is not fully captured by weekly moderate activity thresholds.

Response: The reviewer is correct. Although most participants accumulated ≥150 min/week of MVPA, ENMO values remained lower than those observed in the reference population. This apparent discrepancy likely reflects the fact that these metrics capture different dimensions of physical activity behavior. In our cohort, physical activity was characterized by very little vigorous activity and was predominantly accumulated in short, fragmented bouts rather than in sustained periods of structured exercise. Consequently, participants could achieve the recommended amount of MVPA despite exhibiting reduced overall movement volume and altered activity patterns. These findings suggest that the WHO MVPA threshold alone may not adequately capture impairments in physical activity behavior in individuals with post-COVID condition.

We added the following paragraph to the Discussion section (page 16/17):

“The apparent discrepancy between lower ENMO values and the proportion of participants meeting WHO physical activity recommendations reflects that these metrics capture different dimensions of physical behaviour. ENMO represents overall movement volume, whereas the WHO recommendation is based solely on time accumulated above a moderate-intensity threshold. In our cohort, PA was characterized by minimal vigorous activity and was predominantly accumulated in short, fragmented bouts rather than sustained periods of structured exercise. Consequently, participants could meet the recommended amount of MVPA despite reduced overall movement volume and altered activity patterns. These findings highlight that total activity volume, intensity distribution, bout structure, and vigorous activity provide complementary information that is not captured by weekly MVPA thresholds alone. Accordingly, the WHO MVPA threshold may not fully reflect PA patterns in individuals with post-COVID condition.“

The assessment of post-exertional malaise is a limitation. PEM is central to post-COVID-19 condition, but it was assessed with a single item rather than a validated multidimensional instrument. This limitation should be given more weight, especially when discussing physical reconditioning or activity promotion.

Response: We agree that the assessment of PEM is a major limitation of our study. To be transparent about this limitation, we already acknowledged it in the Methods section and have further emphasised it in the Discussion.

Page 8: “We did not assess PEM using a validated instrument, which limits the validity of our evaluation of this complex symptom.”

Further, we extended the paragraph in the Limitations section to give this limitation more weight, as suggested.

Page 19: “Third, we expected to observe associations between PA and PEM severity, which was not the case in our study. Although, we captured the presence and severity of PEM using a single question, we did not apply a validated instrument to assess PEM as a multifaceted construct encompassing physical, cognitive, and affective dimensions, with delayed onset and variable duration and intensity [46]. This limitation may reduce the validity of our assessment and partly explain the absence of an observed association. Given that PEM is considered a cardinal symptom of PCC [45] and may substantially influence activity behaviour and tolerance to exertion, our findings regarding physical activity patterns and potentially modifiable correlates should be interpreted with caution. In particular, our cross-sectional data do not permit conclusions regarding the safety or effectiveness of physical reconditioning or activity promotion strategies in individuals experiencing PEM”.

Please correct minor language and typographical issues. For example, “accelerometery” should be “accelerometry”, “modelsfor” should be separated, “literature suggest” should be “literature suggests”, and “age- and se-specific” appears to be a typographical error.

Response: Done, thank you.

Overall, this is a valuable and technically sound study. With these minor revisions, the manuscript would provide a clear and useful contribution to the literature on physical activity in post-COVID-19 condition.

Response: Thank you.

Attachment Submitted filename: Response_to_Reviewers_auresp_2.docx

# Editor report: Decision Letter 2

**DOI:** 10.1371/journal.pone.0357465.r005

**Authors:** LS Katrina Li

12 Aug 2026

Dear Dr. Radtke,

Thank you for submitting your manuscript to PLOS ONE. After careful consideration, we feel that it has merit but does not fully meet PLOS ONE’s publication criteria as it currently stands. Therefore, we invite you to submit a revised version of the manuscript that addresses the points raised during the review process.

**ACADEMIC EDITOR:**

Please submit your revised manuscript by Sep 26 2026 11:59PM. If you will need more time than this to complete your revisions, please reply to this message or contact the journal office at plosone@plos.org. When you're ready to submit your revision, log on to https://www.editorialmanager.com/pone/ and select the 'Submissions Needing Revision' folder to locate your manuscript file.

- A letter that responds to each point raised by the academic editor and reviewer(s). You should upload this letter as a separate file labeled 'Response to Reviewers'.
- A marked-up copy of your manuscript that highlights changes made to the original version. You should upload this as a separate file labeled 'Revised Manuscript with Track Changes'.
- An unmarked version of your revised paper without tracked changes. You should upload this as a separate file labeled 'Manuscript'.

If you would like to make changes to your financial disclosure, please include your updated statement in your cover letter. Guidelines for resubmitting your figure files are available below the reviewer comments at the end of this letter.

If applicable, we recommend that you deposit your laboratory protocols in protocols.io to enhance the reproducibility of your results. Protocols.io assigns your protocol its own identifier (DOI) so that it can be cited independently in the future. For instructions see: https://journals.plos.org/plosone/s/submission-guidelines#loc-laboratory-protocols. Additionally, PLOS ONE offers an option for publishing peer-reviewed Lab Protocol articles, which describe protocols hosted on protocols.io. Read more information on sharing protocols at https://plos.org/protocols?utm_medium=editorial-email&utm_source=authorletters&utm_campaign=protocols.

As the corresponding author, your ORCID iD is verified in the submission system and will appear in the published article. PLOS supports the use of ORCID, and we encourage all coauthors to register for an ORCID iD and use it as well. Please encourage your coauthors to verify their ORCID iD within the submission system before final acceptance, as unverified ORCID iDs will not appear in the published article. *Only*  the individual author can complete the verification step; PLOS staff *cannot*  verify ORCID iDs on behalf of authors.

We look forward to receiving your revised manuscript.

Kind regards,

LS Katrina Li

Academic Editor

PLOS One

Journal Requirements:

If the reviewer comments include a recommendation to cite specific previously published works, please review and evaluate these publications to determine whether they are relevant and should be cited. There is no requirement to cite these works unless the editor has indicated otherwise.

Please review your reference list to ensure that it is complete and correct. If you have cited papers that have been retracted, please include the rationale for doing so in the manuscript text, or remove these references and replace them with relevant current references. Any changes to the reference list should be mentioned in the rebuttal letter that accompanies your revised manuscript. If you need to cite a retracted article, indicate the article’s retracted status in the References list and also include a citation and full reference for the retraction notice.

[Note: HTML markup is below. Please do not edit.]

[NOTE: If reviewer comments were submitted as an attachment file, they will be attached to this email and accessible via the submission site. Please log into your account, locate the manuscript record, and check for the action link "View Attachments". If this link does not appear, there are no attachment files.]

To ensure your figures meet our technical requirements, please review our figure guidelines: https://journals.plos.org/plosone/s/figures

You may also use PLOS’s free figure tool, NAAS, to help you prepare publication quality figures: https://journals.plos.org/plosone/s/figures#loc-tools-for-figure-preparation.

NAAS will assess whether your figures meet our technical requirements by comparing each figure against our figure specifications.

# Author comment: Author response to Decision Letter 3

**DOI:** 10.1371/journal.pone.0357465.r006

14 Aug 2026

Dear Dr LS Katrina Li

Thank you very much for your careful review.

We have cited Figure S1 in the revised manuscript on page 9.

Kind regards,

Thomas Radtke

Attachment Submitted filename: Response to Reviewer.docx

# Editor report: Decision Letter 3

**DOI:** 10.1371/journal.pone.0357465.r007

**Authors:** LS Katrina Li

18 Aug 2026

Physical activity in post-COVID-19 condition: a cross-sectional study

PONE-D-25-64893R3

Dear Dr. Radtke,

We’re pleased to inform you that your manuscript has been judged scientifically suitable for publication and will be formally accepted for publication once it meets all outstanding technical requirements.

Within one week, you’ll receive an e-mail detailing the required amendments. When these have been addressed, you’ll receive a formal acceptance letter and your manuscript will be scheduled for publication.

An invoice will be generated when your article is formally accepted. Please note, if your institution has a publishing partnership with PLOS and your article meets the relevant criteria, all or part of your publication costs will be covered. Please make sure your user information is up-to-date by logging into Editorial Manager at Editorial Manager® and clicking the ‘Update My Information' link at the top of the page. For questions related to billing, please contact billing support.

If your institution or institutions have a press office, please notify them about your upcoming paper to help maximize its impact. If they’ll be preparing press materials, please inform our press team as soon as possible -- no later than 48 hours after receiving the formal acceptance. Your manuscript will remain under strict press embargo until 2 pm Eastern Time on the date of publication. For more information, please contact onepress@plos.org.

Kind regards,

LS Katrina Li

Academic Editor

PLOS One

Additional Editor Comments (optional):

Reviewers' comments:

# Editor report: Acceptance letter

**DOI:** 10.1371/journal.pone.0357465.r008

**Authors:** LS Katrina Li

PONE-D-25-64893R3

PLOS One

Dear Dr. Radtke,

I'm pleased to inform you that your manuscript has been deemed suitable for publication in PLOS One. Congratulations! Your manuscript is now being handed over to our production team.

At this stage, our production department will prepare your paper for publication. This includes ensuring the following:

* All references, tables, and figures are properly cited

* All relevant supporting information is included in the manuscript submission,

* There are no issues that prevent the paper from being properly typeset

You will receive further instructions from the production team, including instructions on how to review your proof when it is ready. Please keep in mind that we are working through a large volume of accepted articles, so please give us a few days to review your paper and let you know the next and final steps.

Lastly, if your institution or institutions have a press office, please let them know about your upcoming paper now to help maximize its impact. If they'll be preparing press materials, please inform our press team within the next 48 hours. Your manuscript will remain under strict press embargo until 2 pm Eastern Time on the date of publication. For more information, please contact onepress@plos.org.

You will receive an invoice from PLOS for your publication fee after your manuscript has reached the completed accept phase. If you receive an email requesting payment before acceptance or for any other service, this may be a phishing scheme. Learn how to identify phishing emails and protect your accounts at https://explore.plos.org/phishing.

If we can help with anything else, please email us at customercare@plos.org.

Thank you for submitting your work to PLOS One and supporting open access.

Kind regards,

PLOS One Editorial Office Staff

on behalf of

Dr. LS Katrina Li

Academic Editor

PLOS One
